# Supplementary figures and images for: Households’ poverty and inequality after the COVID-19: Insights from panel data of face-to-face surveys in Southeast Asia
Source: PLoS One. 2026 Jan 30;21(1):e0341648. doi: 10.1371/journal.pone.0341648 (PMC12922772; doi:10.1371/journal.pone.0341648)

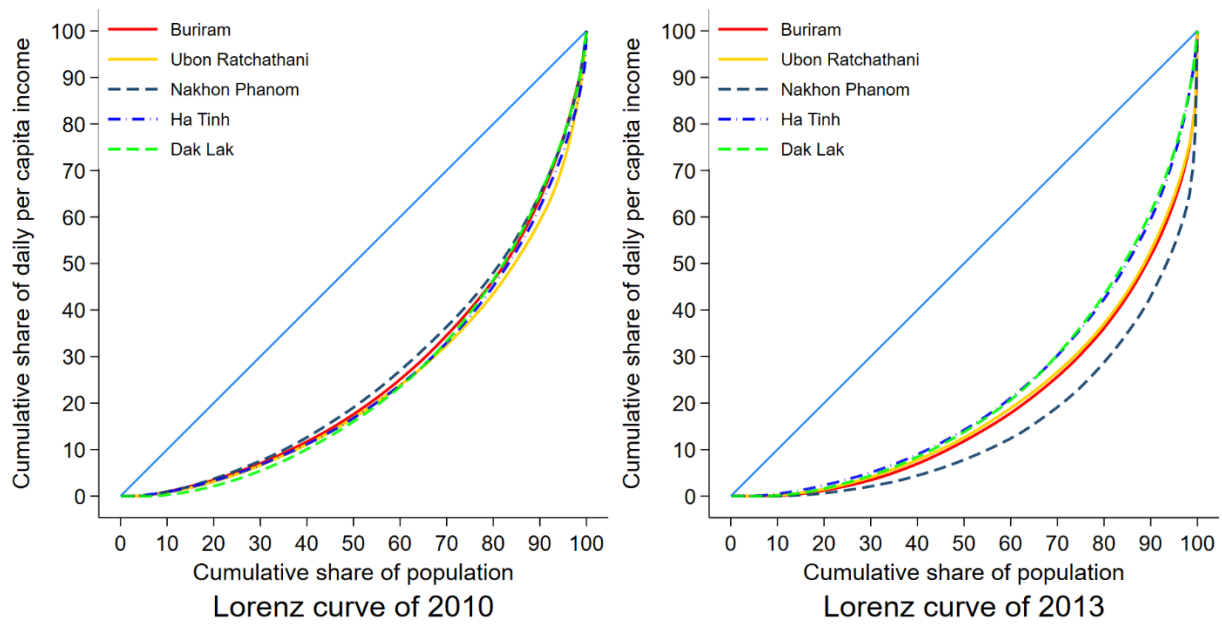

**S1 Fig. Lorenz curves of daily per capita income in each province in 2010 and 2013**

Supplement: S1 Fig — (PDF) [file pone.0341648.s001.pdf]
