# Supplementary material for: Households’ poverty and inequality after the COVID-19: Insights from panel data of face-to-face surveys in Southeast Asia
Source: PLoS One. 2026 Jan 30;21(1):e0341648. doi: 10.1371/journal.pone.0341648 (PMC12922772; doi:10.1371/journal.pone.0341648)
Supplement: S1 Table — (PDF) [file pone.0341648.s002.pdf]

S1 Table. Name, definition and measurement of variables

Variables	Measurement	Definition
A. Household income		
Daily per capita income	PPP$ (adjusted to 2005 prices)	Daily per capita income of the household in the past 12 months
B. Household characteristics		
A member contracted to the COVID-19	Dummy	The household has at least a member contracted to the COVID-19 in the past 12 months = 1; otherwise = 0
Age of household head	Years of age	Age of the household head
Gender of head	Dummy	The household is male-headed = 1; otherwise = 0
Ethnicity of head	Dummy	The household belongs to Thai majority (Thailand) or Kinh majority (Vietnam) = 1; otherwise = 0
Household size	Number of persons	Number of nucleus members in the household
Number of adults	Number of persons	Number of adult members (older than 18 and not older than 60 years old) in the household
Number of elderly members	Number of persons	Number of elderly members (older than 60 years old) in the household
Member of political and social organization (PSO)	Dummy	The household head is a member of political and social organization = 1; otherwise = 0
Share of farm labourers	Percentage (%)	The share of household members who are working in farming activities
Schooling years of household head	Years of schooling	Number of schooling years of the household head
Mean schooling years of adult members	Years of schooling	Average schooling years of adult members in the household
Experienced a shock in the past 12 months	Dummy	If the household experienced a shock in the past 12 months = 1; otherwise = 0
Household land area per capita	hectares (ha)	Total land area per capita of the household
Household asset value per capita	PPP$ (adjusted to 2005 prices)	Total asset values per capita of the household including productive and non-productive assets
C. Multidimensional poverty parameters		
Income poverty	Dummy	Daily income per capita of the household is at or lower than PPP$ 3.20 = 1; otherwise = 0
No schooling of school-age children	Dummy	The household has at least one school-age child up to the grade-8 age not enrolling in school = 1; otherwise = 0
No primary education of adult members	Dummy	The household has no adults at the grade-9 age or above completed a primary education = 1; otherwise = 0
Unsafe drinking water	Dummy	Drinking water of the household comes from unsafe sources (river, lake, pond…) = 1; otherwise = 0
No improved sanitation	Dummy	There is no flush toilet in the household = 1; otherwise = 0
No access to electricity	Dummy	There is no access to electricity for lighting in the household = 1; otherwise = 0
D. Province's socio-economic indicators		
Unemployment rate	Percentage (%)	The share of people in working ages having no employment in the province
Share of rural population	Percentage (%)	The share of the population living in rural areas of the province
