# Supplementary material for: Households’ poverty and inequality after the COVID-19: Insights from panel data of face-to-face surveys in Southeast Asia
Source: PLoS One. 2026 Jan 30;21(1):e0341648. doi: 10.1371/journal.pone.0341648 (PMC12922772; doi:10.1371/journal.pone.0341648)
Supplement: S2 Table — (PDF) [file pone.0341648.s003.pdf]

S2 Table. The adopted and adjusted measure of multidimensional poverty

Dimension	Parameter	Weight
Monetary dimension	Daily per capita income of the household is at or lower than PPP$ 3.20	1/3
Educational dimension	The household has at least one school-age child up to the grade-8 age not enrolling in school	1/6
	The household has no adults at the grade-9 age or above completed a primary education	1/6
Access to basic infrastructure	Drinking water of the household comes from unsafe sources	1/9
	There is no improved sanitation (flush toilet) in the household	1/9
	There is no access to electricity for lighting in the household	1/9
