# Supplementary material for: Households’ poverty and inequality after the COVID-19: Insights from panel data of face-to-face surveys in Southeast Asia
Source: PLoS One. 2026 Jan 30;21(1):e0341648. doi: 10.1371/journal.pone.0341648 (PMC12922772; doi:10.1371/journal.pone.0341648)
Supplement: S3 Table — (PDF) [file pone.0341648.s004.pdf]

S3 Table. Variance inflation factor (VIF) values of independent variables

Variables	Without interactions (1)	With interactions		
		(2)	(3)	(4)
COVID-19 period†	1.83	4.23	6.03	8.30
COVID-19*Male head		3.70		
COVID-19*Share of farm laborers			4.85	
COVID-19*Mean schooling years of adults				9.54
Member contracted to the COVID-19†	1.38	1.38	1.39	1.42
Age of head	2.40	2.41	2.40	2.40
Male head†	1.13	1.55	1.13	1.13
Ethnic majority†	1.16	1.16	1.16	1.16
Household size	2.78	2.78	2.78	2.78
Number of adults	3.28	3.28	3.28	3.28
Number of elderly members	3.13	3.13	3.13	3.13
PSO member†	1.53	1.53	1.53	1.53
Share of farm laborers	1.11	1.11	1.59	1.13
Schooling years of head	1.87	1.87	1.87	1.88
Mean schooling years of adult members	1.68	1.68	1.70	2.03
Shock exposure†	1.08	1.08	1.08	1.08
Land area per capita	1.23	1.23	1.23	1.23
Asset poor†	1.11	1.11	1.11	1.11
Province's unemployment rate	1.60	1.60	1.60	1.60
Province's share of rural population	1.06	1.06	1.06	1.07
**Mean VIF**	**1.73**	**1.99**	**2.16**	**2.54**

Note: †: Dummy
