# Supplementary material for: Households’ poverty and inequality after the COVID-19: Insights from panel data of face-to-face surveys in Southeast Asia
Source: PLoS One. 2026 Jan 30;21(1):e0341648. doi: 10.1371/journal.pone.0341648 (PMC12922772; doi:10.1371/journal.pone.0341648)
Supplement: S4 Table — (PDF) [file pone.0341648.s005.pdf]

S4 Table. Heterogeneous effects of the COVID-19 on household income, Gini coefficient, and poverty (Fixed-effects estimations): The case of head gender

	Daily per capita income (ln)	Gini coefficient of household income	Income poverty at PPP$ 3.20†	Multidimensional poverty†
COVID-19 period†	-0.258***	0.009***	0.102***	0.105***
	(0.095)	(0.003)	(0.024)	(0.024)
COVID-19*Male head	0.056	0.005**	-0.045*	-0.049**
	(0.089)	(0.002)	(0.024)	(0.024)
Member contracted to the COVID-19†	-0.084	0.007***	-0.012	-0.016
	(0.092)	(0.002)	(0.022)	(0.022)
Age of head	0.017***	0.000	-0.003***	-0.003***
	(0.004)	(0.000)	(0.001)	(0.001)
Male head†	-0.162*	0.007**	0.033	0.038
	(0.093)	(0.003)	(0.026)	(0.025)
Ethnic majority†	0.108	-0.007	-0.031	-0.033
	(0.180)	(0.006)	(0.045)	(0.045)
Household size	-0.236***	0.001*	0.089***	0.090***
	(0.025)	(0.001)	(0.007)	(0.007)
Number of adults	0.218***	-0.000	-0.092***	-0.091***
	(0.036)	(0.001)	(0.009)	(0.009)
Number of elderly members	0.174***	-0.004**	-0.086***	-0.086***
	(0.054)	(0.002)	(0.014)	(0.014)
PSO member†	-0.023	-0.000	0.003	0.009
	(0.073)	(0.002)	(0.015)	(0.015)
Share of farm laborers	-0.004***	0.000***	0.001***	0.001***
	(0.001)	(0.000)	(0.000)	(0.000)
Schooling years of head	0.035***	-0.003***	-0.006*	-0.006*
	(0.013)	(0.000)	(0.003)	(0.003)
Mean schooling years of adult members	0.036***	0.002***	-0.012***	-0.012***
	(0.010)	(0.000)	(0.002)	(0.002)
Shock exposure	-0.086**	-0.010***	0.010	0.011
	(0.043)	(0.002)	(0.011)	(0.011)
Land area per capita	0.012	0.013***	-0.032***	-0.031***
	(0.058)	(0.001)	(0.009)	(0.009)
Asset poor†	-0.239***	0.002	0.091***	0.088***
	(0.057)	(0.002)	(0.014)	(0.014)
Province's unemployment rate	-0.094***	0.012***	0.022***	0.022***
	(0.025)	(0.001)	(0.006)	(0.006)
Province's share of rural population	-0.022***	0.006***	0.005**	0.005**
	(0.008)	(0.001)	(0.002)	(0.002)
Constant	2.386***	0.025	0.109	0.100
	(0.682)	(0.052)	(0.194)	(0.191)
Number of observations	10068	10068	10068	10068
F(18,361)	15.001	24.102	24.979	24.356
Prob. > F	0.000	0.000	0.000	0.000
R^2:				
Within	0.032	0.182	0.053	0.053
Between	0.086	0.389	0.158	0.176
Overall	0.048	0.233	0.093	0.101

Note: Robust standard errors clustered at village level in parentheses; †: Dummy; ln: natural logarithm; *** p<0.01, ** p<0.05, * p<0.1.
