# Supplementary material for: Households’ poverty and inequality after the COVID-19: Insights from panel data of face-to-face surveys in Southeast Asia
Source: PLoS One. 2026 Jan 30;21(1):e0341648. doi: 10.1371/journal.pone.0341648 (PMC12922772; doi:10.1371/journal.pone.0341648)
Supplement: S5 Table — (PDF) [file pone.0341648.s006.pdf]

S5 Table. Heterogeneous effects of the COVID-19 on household income, Gini coefficient, and poverty (Fixed-effects estimations): The case of the share of farm laborers

	Daily per capita income (ln)	Gini coefficient of household income	Income poverty at PPP$ 3.20†	Multidimensional poverty†
COVID-19 period†	0.033	0.019***	0.033	0.036
	(0.104)	(0.004)	(0.029)	(0.028)
COVID-19*Share of farm laborers	-0.004***	-0.000**	0.001	0.000
	(0.001)	(0.000)	(0.000)	(0.000)
Member contracted to the COVID-19†	-0.102	0.006**	-0.010	-0.015
	(0.092)	(0.002)	(0.022)	(0.022)
Age of head	0.017***	0.000	-0.004***	-0.004***
	(0.004)	(0.000)	(0.001)	(0.001)
Male head†	-0.133	0.010***	0.013	0.016
	(0.089)	(0.003)	(0.023)	(0.023)
Ethnic majority†	0.111	-0.007	-0.029	-0.030
	(0.179)	(0.006)	(0.045)	(0.044)
Household size	-0.235***	0.001*	0.089***	0.090***
	(0.026)	(0.001)	(0.007)	(0.007)
Number of adults	0.217***	-0.000	-0.092***	-0.092***
	(0.036)	(0.001)	(0.009)	(0.009)
Number of elderly members	0.180***	-0.004**	-0.088***	-0.088***
	(0.055)	(0.002)	(0.014)	(0.014)
PSO member†	-0.024	-0.000	0.005	0.011
	(0.074)	(0.002)	(0.015)	(0.015)
Share of farm laborers	-0.003***	0.000***	0.001***	0.001***
	(0.001)	(0.000)	(0.000)	(0.000)
Schooling years of head	0.035***	-0.003***	-0.006*	-0.006*
	(0.012)	(0.000)	(0.003)	(0.003)
Mean schooling years of adult members	0.032***	0.002***	-0.011***	-0.011***
	(0.010)	(0.000)	(0.002)	(0.002)
Shock exposure	-0.086**	-0.010***	0.010	0.010
	(0.043)	(0.002)	(0.011)	(0.011)
Land area per capita	0.012	0.013***	-0.032***	-0.032***
	(0.058)	(0.001)	(0.009)	(0.009)
Asset poor†	-0.235***	0.002	0.090***	0.087***
	(0.056)	(0.002)	(0.014)	(0.014)
Province's unemployment rate	-0.099***	0.012***	0.023***	0.023***
	(0.025)	(0.001)	(0.006)	(0.006)
Province's share of rural population	-0.022***	0.006***	0.005**	0.005**
	(0.008)	(0.001)	(0.002)	(0.002)
Constant	2.281***	0.023	0.127	0.116
	(0.686)	(0.052)	(0.194)	(0.191)
Number of observations	10068	10068	10068	10068
$F(18,361)$	14.876	24.101	25.209	24.593
Prob. > F	0.000	0.000	0.000	0.000
R^2:				
Within	0.032	0.183	0.053	0.053
Between	0.081	0.391	0.152	0.169
Overall	0.047	0.234	0.091	0.098

Note: Robust standard errors clustered at village level in parentheses; †: Dummy; ln: natural logarithm; *** p<0.01, ** p<0.05, * p<0.1.
