# Supplementary material for: Households’ poverty and inequality after the COVID-19: Insights from panel data of face-to-face surveys in Southeast Asia
Source: PLoS One. 2026 Jan 30;21(1):e0341648. doi: 10.1371/journal.pone.0341648 (PMC12922772; doi:10.1371/journal.pone.0341648)
Supplement: S6 Table — (PDF) [file pone.0341648.s007.pdf]

S6 Table. Heterogeneous effects of the COVID-19 on household income, Gini coefficient, and poverty (Fixed-effects estimations): The case of the education of adult members

	Daily per capita income (ln)	Gini coefficient of household income	Income poverty at PPP$ 3.20†	Multidimensional poverty†
COVID-19 period†	-0.401***	-0.010***	0.193***	0.190***
	(0.134)	(0.004)	(0.033)	(0.034)
COVID-19*Mean schooling years of adults	0.027*	0.003***	-0.018***	-0.018***
	(0.016)	(0.000)	(0.004)	(0.004)
Member contracted to the COVID-19†	-0.111	0.003	0.006	0.001
	(0.092)	(0.002)	(0.022)	(0.022)
Age of head	0.017***	0.000	-0.003***	-0.003***
	(0.004)	(0.000)	(0.001)	(0.001)
Male head†	-0.133	0.010***	0.011	0.014
	(0.090)	(0.003)	(0.023)	(0.023)
Ethnic majority†	0.102	-0.007	-0.027	-0.028
	(0.180)	(0.006)	(0.045)	(0.044)
Household size	-0.235***	0.002**	0.089***	0.089***
	(0.026)	(0.001)	(0.007)	(0.007)
Number of adults	0.218***	-0.000	-0.092***	-0.092***
	(0.036)	(0.001)	(0.009)	(0.009)
Number of elderly members	0.174***	-0.004***	-0.086***	-0.086***
	(0.054)	(0.002)	(0.014)	(0.014)
PSO member†	-0.024	-0.000	0.004	0.010
	(0.073)	(0.002)	(0.015)	(0.015)
Share of farm laborers	-0.004***	0.000***	0.001***	0.001***
	(0.001)	(0.000)	(0.000)	(0.000)
Schooling years of head	0.033***	-0.003***	-0.004	-0.005
	(0.013)	(0.000)	(0.003)	(0.003)
Mean schooling years of adult members	0.027**	0.001**	-0.006**	-0.006**
	(0.011)	(0.000)	(0.003)	(0.003)
Shock exposure	-0.085**	-0.010***	0.010	0.011
	(0.043)	(0.002)	(0.011)	(0.011)
Land area per capita	0.010	0.012***	-0.031***	-0.031***
	(0.058)	(0.001)	(0.009)	(0.009)
Asset poor†	-0.240***	0.002	0.092***	0.089***
	(0.056)	(0.002)	(0.014)	(0.014)
Province's unemployment rate	-0.095***	0.012***	0.023***	0.022***
	(0.025)	(0.001)	(0.006)	(0.006)
Province's share of rural population	-0.021***	0.006***	0.004*	0.004*
	(0.008)	(0.001)	(0.002)	(0.002)
Constant	2.340***	0.020	0.140	0.130
	(0.683)	(0.051)	(0.193)	(0.191)
Number of observations	10068	10068	10068	10068
F(18,361)	14.967	26.674	27.283	26.523
Prob. > F	0.000	0.000	0.000	0.000
R^2:				
Within	0.032	0.188	0.055	0.055
Between	0.085	0.392	0.155	0.172
Overall	0.048	0.237	0.093	0.100

Note: Robust standard errors clustered at village level in parentheses; †: Dummy; ln: natural logarithm; *** p<0.01, ** p<0.05, * p<0.1.
