# Supplementary material for: Households’ poverty and inequality after the COVID-19: Insights from panel data of face-to-face surveys in Southeast Asia
Source: PLoS One. 2026 Jan 30;21(1):e0341648. doi: 10.1371/journal.pone.0341648 (PMC12922772; doi:10.1371/journal.pone.0341648)
Supplement: S7 Table — (PDF) [file pone.0341648.s008.pdf]

S7 Table. Distributional effects of the COVID-19 on household income

	Daily per capita income (PPP$)				
	10th	25th	50th	75th	90th
COVID-19 period†	-0.385***	-0.448***	-0.487***	-0.122	-0.305
	(0.096)	(0.106)	(0.158)	(0.300)	(0.725)
Member contracted to the COVID-19†	-0.251*	0.058	0.351*	0.774*	1.878
	(0.141)	(0.138)	(0.213)	(0.411)	(1.179)
Age of head	0.003	-0.007	-0.012*	-0.011	0.023
	(0.004)	(0.004)	(0.006)	(0.011)	(0.026)
Male head†	0.103	0.219**	0.277**	0.393	0.338
	(0.071)	(0.090)	(0.125)	(0.240)	(0.619)
Ethnic majority†	0.203	0.324**	0.467**	-0.229	-0.801
	(0.128)	(0.144)	(0.203)	(0.316)	(0.688)
Household size	-0.163***	-0.367***	-0.835***	-1.608***	-2.634***
	(0.029)	(0.035)	(0.054)	(0.092)	(0.240)
Number of adults	0.183***	0.414***	0.944***	1.619***	2.168***
	(0.042)	(0.051)	(0.077)	(0.132)	(0.338)
Number of elderly members	0.203***	0.385***	0.743***	1.036***	0.410
	(0.061)	(0.076)	(0.120)	(0.198)	(0.462)
PSO member†	0.078	0.150*	0.153	0.770***	0.784
	(0.079)	(0.089)	(0.138)	(0.241)	(0.698)
Share of farm laborers	-0.006***	-0.010***	-0.020***	-0.034***	-0.061***
	(0.001)	(0.001)	(0.002)	(0.003)	(0.009)
Schooling years of head	0.010	0.036***	0.103***	0.298***	0.766***
	(0.011)	(0.012)	(0.021)	(0.042)	(0.126)
Mean schooling years of adult members	0.056***	0.108***	0.240***	0.423***	0.822***
	(0.012)	(0.014)	(0.023)	(0.046)	(0.121)
Shock exposure†	-0.235***	-0.285***	-0.294***	-0.479**	-0.734
	(0.057)	(0.068)	(0.101)	(0.198)	(0.495)
Land area per capita	0.030	0.236***	0.610***	1.572***	4.114***
	(0.040)	(0.045)	(0.073)	(0.143)	(0.465)
Asset poor†	-0.614***	-1.305***	-2.250***	-2.653***	-3.638***
	(0.091)	(0.099)	(0.136)	(0.220)	(0.460)
Province’s unemployment rate	-0.058*	-0.130***	-0.249***	-0.535***	-0.706***
	(0.031)	(0.036)	(0.055)	(0.101)	(0.252)
Province’s share of rural population	-0.024***	-0.043***	-0.058***	-0.089***	-0.219***
	(0.008)	(0.009)	(0.015)	(0.028)	(0.075)
Constant	2.919***	6.091***	9.701***	16.276***	30.297***
	(0.721)	(0.799)	(1.295)	(2.409)	(6.284)
Number of observations	10068	10068	10068	10068	10068
F (17,361)	13.540	48.977	142.417	88.267	26.218
Prob. > F	0.000	0.000	0.000	0.000	0.000
R^2	0.032	0.095	0.154	0.153	0.098
Sample mean RIF	0.859	2.000	4.202	8.007	14.341

Note: Robust standard errors clustered at village level in parentheses; †: Dummy; *** p<0.01, ** p<0.05, * p<0.1.
