# Supplementary material for: Households’ poverty and inequality after the COVID-19: Insights from panel data of face-to-face surveys in Southeast Asia
Source: PLoS One. 2026 Jan 30;21(1):e0341648. doi: 10.1371/journal.pone.0341648 (PMC12922772; doi:10.1371/journal.pone.0341648)
Supplement: S8 Table — (PDF) [file pone.0341648.s009.pdf]

S8 Table. Robustness check of the distributional effects of the COVID-19 on household income using balancing weights

	Daily per capita income (PPP$)				
	10th	25th	50th	75th	90th
COVID-19 period†	-0.771**	-1.003***	-0.976*	0.385	-5.131***
	(0.355)	(0.377)	(0.519)	(1.120)	(1.258)
Member contracted to the COVID-19†	-0.115	0.128	-2.795*	-3.038	2.626
	(0.567)	(0.592)	(1.507)	(2.184)	(2.550)
Age of head	0.016	0.004	-0.025	-0.030	0.116***
	(0.013)	(0.019)	(0.016)	(0.037)	(0.043)
Male head†	0.280	0.309	0.429	0.374	0.648
	(0.177)	(0.231)	(0.282)	(0.394)	(0.892)
Ethnic majority†	-0.110	0.373	0.944***	0.408	0.017
	(0.362)	(0.500)	(0.279)	(0.341)	(0.906)
Household size	-0.218*	-0.323***	-0.708***	-0.924***	-2.419***
	(0.119)	(0.083)	(0.098)	(0.181)	(0.314)
Number of adults	0.228	0.486***	1.078***	1.670***	2.183***
	(0.167)	(0.127)	(0.141)	(0.273)	(0.526)
Number of elderly members	0.202	0.461*	0.997***	1.536**	-0.297
	(0.266)	(0.270)	(0.321)	(0.711)	(0.770)
PSO member†	0.537***	0.676***	0.851*	1.479*	-2.964**
	(0.191)	(0.238)	(0.459)	(0.869)	(1.194)
Share of farm laborers	-0.009***	-0.014***	-0.025***	-0.025***	-0.058***
	(0.003)	(0.003)	(0.004)	(0.007)	(0.012)
Schooling years of head	0.025	0.056**	0.134***	0.279***	0.767***
	(0.033)	(0.028)	(0.048)	(0.093)	(0.187)
Mean schooling years of adult members	0.046	0.072**	0.218***	0.182**	0.538***
	(0.030)	(0.030)	(0.045)	(0.071)	(0.151)
Shock exposure†	-0.131	-0.059	0.132	-0.356	-0.788
	(0.159)	(0.195)	(0.268)	(0.481)	(0.691)
Land area per capita	0.063	0.376***	0.818***	1.520***	4.011***
	(0.077)	(0.093)	(0.120)	(0.229)	(0.538)
Asset poor†	-0.674***	-1.081***	-2.330***	-2.146***	-3.029***
	(0.239)	(0.258)	(0.219)	(0.294)	(0.658)
Province's unemployment rate	-0.144*	-0.218**	-0.497***	-0.614**	0.201
	(0.081)	(0.086)	(0.143)	(0.291)	(0.406)
Province's share of rural population	-0.041**	-0.037***	-0.041*	-0.036	-0.095
	(0.016)	(0.013)	(0.021)	(0.031)	(0.085)
Constant	3.812**	4.558***	7.722***	10.060***	15.135**
	(1.649)	(1.341)	(2.053)	(3.127)	(7.655)
Number of observations	10068	10068	10068	10068	10068
F (17,361)	4.005	15.099	83.176	56.853	20.437
Prob. > F	0.000	0.000	0.000	0.000	0.000
R^2	0.075	0.130	0.200	0.177	0.096
Sample mean RIF	0.693	1.773	3.931	7.803	12.432
Impact magnitude on household's daily per capita income	-111.33%	-56.58%	-24.83%	Not significant	-41.27%

Note: Robust standard errors clustered at village level in parentheses; †: Dummy; *** p<0.01, ** p<0.05, * p<0.1.
