# Supplementary material for: Households’ poverty and inequality after the COVID-19: Insights from panel data of face-to-face surveys in Southeast Asia
Source: PLoS One. 2026 Jan 30;21(1):e0341648. doi: 10.1371/journal.pone.0341648 (PMC12922772; doi:10.1371/journal.pone.0341648)
Supplement: Attachment — Submitted filename: comments_plos1.docx [file pone.0341648.s011.docx]

**Households’ poverty and inequality after the COVID-19: Insights from panel data of**

**face-to-face surveys in Southeast Asia**

The paper makes a valuable contribution by using robust panel data and appropriate econometric techniques to analyze the multifaceted impacts of COVID-19 on rural households in Thailand and Vietnam. The findings on increased poverty, inequality, and the heterogeneous/distributional effects are largely compelling. However, there are a few critical points, particularly concerning the Gini coefficient analysis and some discrepancies in reported results, that need attention.

1. Page 7, text description of asset value: "when it drops from PPP 769 between 2010 and 2016 to only PPP 78 between 2016 and 2022." This refers to the increase in asset value during those periods, not the asset value itself. Phrasing could be clearer to avoid misinterpretation. Moreover, this mean difference is not significant.
2. A member contracted to the COVID-19 (yes = 1)" variable (Table 1 & 4), Page 6 states "about 31% of rural households in our sample having a member contracted to the COVID-19 in 2022." This figure seems high for actual infection rates in a broad rural sample, even during peak waves. Could this variable be capturing households reporting a member contracted, or perhaps a broader definition of being directly affected by illness? Clarification on how this was measured would be beneficial.
3. In Table 4, this variable's coefficient for income is -0.092 but not statistically significant. Its effect on the Gini coefficient is negative and significant (-0.010), which is an interesting finding if the Gini is at the provincial level (discussed under Methodology).
4. **Gini Coefficient (Table 2 & as dependent variable in Table 4):**

A Gini coefficient of 0.90 for Nakhon Phanom in 2022 (Table 2) is extraordinarily high, bordering on perfect inequality. While possible, such an extreme value warrants a double-check of the calculation or a brief comment on potential drivers or data particularities for that province-year.

The more significant concern is its use as a household-level dependent variable (Y_iw) in Table 4. A Gini coefficient is an aggregate measure of inequality for a population (e.g., a province or village). It's not a household-level attribute.

If it's the province-level Gini: The unit of analysis for this dependent variable is province-wave. The regression in Table 4 lists N=10084 (household-wave observations). This implies each household within a province-wave is assigned the same Gini value. While this is sometimes done, it artificially inflates N for the Gini regression and standard errors need very careful handling (clustering at province level would be essential, village level might not be sufficient if Gini is province-level). The interpretation of household-level X_iw predicting a province-level Gini also becomes nuanced.

If it's some non-standard household-level Gini: This would require extensive justification and explanation, as it's not a conventional measure. This is the most critical methodological point to address.

5. Ensure Consistency in Reported Results: Correct the discrepancies in percentage/point changes between the main text summary (page 20) and the regression tables (Table 4).
6. Correct Interpretation of Education Interaction: Ensure the text on page 17 accurately reflects that education *reduces* the poverty impact of COVID-19, consistent with Table 5.
