# Supplementary material for: Households’ poverty and inequality after the COVID-19: Insights from panel data of face-to-face surveys in Southeast Asia
Source: PLoS One. 2026 Jan 30;21(1):e0341648. doi: 10.1371/journal.pone.0341648 (PMC12922772; doi:10.1371/journal.pone.0341648)
Supplement: Attachment — Submitted filename: 2. Response_to_editor_reviewers.docx [file pone.0341648.s013.docx]

**Response to the Editor and Reviewers’ comments and suggestions**

*Please note: the questions, comments, and suggestions of the Editor and reviewers are in blue, our responses in black, and the revised/newly added text in red.*

**1. Comments and suggestion from the Editor**

I have now received two reports on your paper. The referees make a number of constructive recommendations for improvement. While I am unable to accept the paper for publication in its current form, I would be pleased to reconsider it if you revise the manuscript carefully in line with the referees’ suggestions. I should emphasize that some of the revisions requested are quite substantial. I hope you will be willing to undertake this additional work, as otherwise I will be unable to give further consideration to the paper.

**Response:**

Thank you very much for your consideration and comments. We have carefully considered and addressed all of your comments. Our detailed response to each of your comment/suggestion can be found below.

1.1 Please include the following items when submitting your revised manuscript:

A rebuttal letter that responds to each point raised by the academic editor and reviewer(s). You should upload this letter as a separate file labeled 'Response to Reviewers'.

A marked-up copy of your manuscript that highlights changes made to the original version. You should upload this as a separate file labeled 'Revised Manuscript with Track Changes'.

An unmarked version of your revised paper without tracked changes. You should upload this as a separate file labeled 'Manuscript'.

**Response:**

Thank you for this thoughtful instruction. In our revised manuscript, we have included a “Response_to_editor_reviewers” file (this current file), a “Revised_manuscript_with_track_changes” file, and a “Manuscript” file as recommended.

1.2 When submitting your revision, we need you to address these additional requirements.

1.2.1 Please ensure that your manuscript meets PLOS ONE's style requirements, including those for file naming.

**Response:**

Thank you for sharing the information. We have revised our manuscript following the formalities of these samples. In particular, we have restructured our manuscript to have (i) Introduction, (ii) Materials and methods, (iii) Results and discussion, and (iv) Conclusions and policy implications.

1.2.2 Thank you for stating the following in the Acknowledgments Section of your manuscript:

“This study relies on data from the long-term project No. 20220831434900116103, funded by the Deutsche Forschungsgemeinschaft (DFG).”

We note that you have provided additional information within the Acknowledgements Section that is not currently declared in your Funding Statement. Please note that funding information should not appear in the Acknowledgments section or other areas of your manuscript. We will only publish funding information present in the Funding Statement section of the online submission form.

Please remove any funding-related text from the manuscript and let us know how you would like to update your Funding Statement. Currently, your Funding Statement reads as follows:

“The author(s) received no specific funding for this work.”

Please include your amended statements within your cover letter; we will change the online submission form on your behalf.

**Response:**

We appreciate your detailed instructions on how to mention the funding. In fact, we do not receive any funding for this research as indicated in the current funding statement as “The author(s) received no specific funding for this work”. The funding information, which we mentioned in the “Acknowledgements”, is required by the project when using the data. The required acknowledgement from this project can be found via the website “https://www.tvsep.de/en/tvsep-data-access”.

Furthermore, we also rely on the previous work published in *Plos One* which used the same dataset as ours to incorporate the project information in the “Acknowledgements” (please see: https://doi.org/10.1371/journal.pone.0317247 for more information).

Hence, we believe that keeping the “Acknowledgements” and “Funding Statement” as in the current form is relevant and reasonable.

3. Please include your full ethics statement in the ‘Methods’ section of your manuscript file. In your statement, please include the full name of the IRB or ethics committee who approved or waived your study, as well as whether or not you obtained informed written or verbal consent. If consent was waived for your study, please include this information in your statement as well.

**Response:**

Thank you for your comment. We have included a full ethics statement in the “Data” sub-section under the “Materials and methods” section. We use the household data provided by the project “Thailand - Vietnam Socioeconomic Panel (TVSEP), [www.tvsep.de](http://www.tvsep.de)). Before the survey started, the local partners of the TVSEP project have obtained the ethical approval as required by local authorities. However, these documents are not available publicly to data users. The data are anonymous and the respondents were verbally asked for their consent to conduct the interview (see <https://www.tvsep.de/en/survey-documents#c68719> for documentations of the data).

***- Revised text: The second paragraph of the “Data” sub-section (Page 5)***

*…For the wave of 2022, the TVSEP project could collect the data from households in all three provinces in Thailand and only two provinces in Vietnam (see https://www.tvsep.de/en/survey-documents for more information about the survey waves and survey instruments). Before the survey started, the local partners of the TVSEP project had obtained the ethical approval as required by local authorities. However, these documents are not available publicly to data users. The data of the TVSEP project are anonymous and the respondents were verbally asked for their consent to conduct the interview (see* [*https://www.tvsep.de/en/survey-documents#c68719*](https://www.tvsep.de/en/survey-documents#c68719) *for documentations of the data).*

4. Please include captions for your Supporting Information files at the end of your manuscript, and update any in-text citations to match accordingly. Please see our Supporting Information guidelines for more information: [http://journals.plos.org/plosone/s/supporting-information](http://track.editorialmanager.com/CL0/http:%2F%2Fjournals.plos.org%2Fplosone%2Fs%2Fsupporting-information/1/010f0199841da47f-7b00f7ca-4981-4949-b129-5b53c5ef36a5-000000/1uDwoHSaqBqvuo4Vbt0fozJ4-llReWoKtHmOwTbqPKg=230).

**Response:**

We thank you for this reminder. In this revised version, all supporting information has been revised/ split into separate files as explained in the guidelines.

5. We note that Figure 1 in your submission contain [map/satellite] images which may be copyrighted. All PLOS content is published under the Creative Commons Attribution License (CC BY 4.0), which means that the manuscript, images, and Supporting Information files will be freely available online, and any third party is permitted to access, download, copy, distribute, and use these materials in any way, even commercially, with proper attribution. For these reasons, we cannot publish previously copyrighted maps or satellite images created using proprietary data, such as Google software (Google Maps, Street View, and Earth). For more information, see our copyright guidelines: http://journals.plos.org/plosone/s/licenses-and-copyright.

**Response:**

Thank you for your helpful comment. The map (Fig 1) that we include in our manuscript owned by one of the co-authors. However, we cannot ensure the permission of the GIS data used to produce the map. Therefore, we agree to remove the map in the manuscript.

**2. Comments and suggestion from the Reviewer #1**

Reviewer #1: The paper makes a valuable contribution by using robust panel data and appropriate econometric techniques to analyze the multifaceted impacts of COVID-19 on rural households in Thailand and Vietnam. The findings on increased poverty, inequality, and the heterogeneous/distributional effects are largely compelling. However, there are a few critical points, particularly concerning the Gini coefficient analysis and some discrepancies in reported results, that need attention.

**Response:**

Thank you very much for your constructive comments and suggestions which helped us improve our manuscript. We have acknowledged your help in the acknowledgements section. As you can see in this revised version, all your comments and suggestions have been carefully considered and addressed. In particularly, your comment on extreme Gini values in 2022 is absolutely true and we have taken action to address the issue as in our response to your comment #4.1. Thank you very much for the comments!

1. Page 7, text description of asset value: "when it drops from PPP 769 between 2010 and 2016 to only PPP 78 between 2016 and 2022." This refers to the increase in asset value during those periods, not the asset value itself. Phrasing could be clearer to avoid misinterpretation. Moreover, this mean difference is not significant.

**Response:**

Thank you for your comment which we follow. We have revised the paragraph related to the description of asset value to clearly explain the differences as you can see as below. It is important to note that the number of observations has slightly changed due to our action to take out observations with extreme income value as explained in our response to your comment #4.1. This has resulted in a slight change of the mean values in the sample.

***- Revised text: Paragraph 3 of the section “Description of data” (Pages 7 and 8)***

*The average schooling years of household heads and adult members in households are about 5.97 years and 6.32 years, respectively. The average percentage of households reported a shock (e.g., any of weathers shock, health shocks, or economic shocks) in the past 12 months is around 55% in our sample. The average land area per capita of rural households in our sample is roughly 0.63 hectare (ha) and this figure was decreasing from 0.63 ha in 2010 to 0.53 ha in 2022. The average asset value per capita of households shows an increasing tendency between 2010 and 2022 (PPP$ 1,167 in 2010 vs. PPP$ 2,016 in 2022).* *In particular, the average asset value per capita increased from PPP$ 1,167 in 2010 to PPP$ 1,934 in 2016. The difference of the asset value between 2010 and 2016 was about PPP$ 767 and statistically significant. However, the difference of the asset value per capita was just PPP$ 81 between 2016 and 2022 (increased from PPP$ 1,934 in 2016 to PPP$ 2,015 in 2022) and it turned into statistically insignificant.*

2. A member contracted to the COVID-19 (yes = 1)" variable (Table 1 & 4), Page 6 states "about 31% of rural households in our sample having a member contracted to the COVID-19 in 2022." This figure seems high for actual infection rates in a broad rural sample, even during peak waves. Could this variable be capturing households reporting a member contracted, or perhaps a broader definition of being directly affected by illness? Clarification on how this was measured would be beneficial.

**Response:**

Thank you for your question. We relied on the questions “v27012a – Was [Member] infected with the COVID-19”, “v27012aa – infected year”, and “v27012ab – infected month” to construct the variable “member contracted to the COVID-19”. The raw numbers of infected members in these households are presented below. If we look at individual levels, the infection rates were just 16% in Thailand and 19% in Vietnam. However, when we look household level, the number of households with at least a member contracted with the COVID-19 might be relatively higher. For instance, many households in our sample have migrated members (i.e., members working in urban cities), these members are more likely to contract with the COVID-19. In particular, 388 households out of 1,386 households in Thailand and 405 out of 1,131 households in Vietnam have at least a member contracted with the COVID-19 in the past 12 months. In short, this variable captures those households with at least a member contracted with the COVID-19, not the other illnesses.

**Table 1. Number of individuals contracted with the COVID-19 in the TVSEP data in 2022.**

| Question: v27012a | No | Yes | Total answered members |
| --- | --- | --- | --- |
| Thailand | 7,362 (84%) | 1,413 (16%) | 8,775 |
| Vietnam | 4,706 (81%) | 1,084 (19%) | 5,790 |

3. In Table 4, this variable's coefficient for income is -0.092 but not statistically significant. Its effect on the Gini coefficient is negative and significant (-0.010), which is an interesting finding if the Gini is at the provincial level (discussed under Methodology).

**Response:**

Thank you very much for pointing out this detail. After revising the sample and our estimation model, the results in Table 4 have changed slightly. The coefficients of this variable are now “-0.082” (still not significant) in the case of per capita income and “0.007” (positive and significant). The extreme outliers of income really have an influence on the Gini coefficient. We find the result is now more reasonable since the infection of COVID-19 might further deepen the income inequality. We have revised the paragraph related to this result.

***- Revised text: Paragraph 3 of subsection “The correlation and the heterogenous effects of the COVID-19 on household’s income, inequality, and poverty” (Pages 17 – 18)***

*We further find that the variable household with a member contracted with has positive and significant correlation with the Gini coefficient of income inequality. This shows a direct influence of the COVID-19 on income inequality at the provincial level. The age of head, numbers of adults and elderly members in the household, schooling years of head and mean schooling years of adult members have positive correlations with household’s daily per capita income, while household size, share of farm laborers, shock exposure, and asset-poor household have negative correlations with the household’s per capita income. The two variables at provincial level, namely unemployment rate and the share of rural population, also have negative correlations with the per capita income of households. Regarding the Gini coefficient of income inequality, we find that households with at least a member contracted with the Covid-19, male-headed households, household size, share of farm laborers, mean schooling years of adult members, land area per capita, province’s unemployment rate and the share of rural population have positive correlations with the Gini coefficient, while number of elderly members, education of heads, and shock exposure have negative correlations with the Gini coefficient. In addition, the age of head, number of adults, number of elderly members, schooling years of head, average schooling years of adult members, and land area per capita have negative associations, while household size, share of farm laborers, asset-poor households, province’s unemployment rate and the share of rural population have positive associations with absolute and multidimensional poverty.*

4.1 Gini Coefficient (Table 2 & as dependent variable in Table 4):
A Gini coefficient of 0.90 for Nakhon Phanom in 2022 (Table 2) is extraordinarily high, bordering on perfect inequality. While possible, such an extreme value warrants a double-check of the calculation or a brief comment on potential drivers or data particularities for that province-year.

**Response:**

We sincerely thank you for this comment. We have checked the income variables again for observations with extreme values. We found four households with extremely high and we could not validate the values from the raw dataset. These observations significantly affect the mean per capita income in 2020 and the Gini coefficient of income inequality. We decided to take out these four households and 16 observations from the sample (i.e., four households in four waves) to ensure the balanced panel. Together with the comment #1 of the Reviewer #2, we have included two additional variables at the provincial level. This action has resulted in re-calculation and re-estimations of all models across the manuscript. However, the results of two heterogenous effects, namely the interactions of the COVID-19 period with (i) shock exposure and (ii) asset-poor households, have turned into insignificant, therefore, we have to remove the estimations of these interactions in this version. Overall, the main results do not change much compared to the previous version.

4.2 The more significant concern is its use as a household-level dependent variable (Y_iw) in Table 4. A Gini coefficient is an aggregate measure of inequality for a population (e.g., a province or village). It's not a household-level attribute.

If it's the province-level Gini: The unit of analysis for this dependent variable is province-wave. The regression in Table 4 lists N=10084 (household-wave observations). This implies each household within a province-wave is assigned the same Gini value. While this is sometimes done, it artificially inflates N for the Gini regression and standard errors need very careful handling (clustering at province level would be essential, village level might not be sufficient if Gini is province-level). The interpretation of household-level X_iw predicting a province-level Gini also becomes nuanced.

If it's some non-standard household-level Gini: This would require extensive justification and explanation, as it's not a conventional measure. This is the most critical methodological point to address.

**Response:**

Thank you for your insightful comments and explanation which we totally agree. The calculation of the Gini coefficient requires a “population” as mentioned in equation (1). We use the province as the “population” in this regard. It is also correct that households within a province-wave is assigned the same Gini value. We have tried to cluster our analysis at the provincial level, however, the number of included covariates exceeds the number of clusters (17 covariates vs. 5 cluster groups) which makes the estimations invalid. Since the TVSEP data is sampled at the village level, estimations clustered at the village level is more appropriate.

Regarding the use of Gini coefficient at province level for the household-level estimations, we always include the estimations on the Gini coefficient with the estimation of income (the indicator used to calculate the Gini coefficient). In revising the model, we have followed the comment from reviewer 2 to include some variables at the provincial level. Anyway, as you have also pointed out, the interpretation of the results regarding the Gini coefficient should be conducted with care. We acknowledge this as one of our limitations.

***- Added text: Paragraph 5 of the “Conclusions and policy implications” (Pages 22 – 23)***

*Although our study has provided some useful insights, it is not free from limitations. First, we use the panel data which have inconsistent time gaps between panel waves. There was a large time gap between panel waves which might not well reflect the short-term change of household’s livelihood before, during, and after the COVID-19. Hence, the use of panels with shorter time gaps is strongly recommended in future studies. Second, although the attrition rate of our sample is low, due to the fact that one province in Vietnam was excluded in the 2022 survey, the results should be interpreted strictly for the reduced sample. Third, the Gini coefficients of income inequality in our model are calculated at the provincial levels, hence the results regarding the correlation of the COVID-19 with the Gini coefficient should be interpreted with caution. Last, even though the impacts of the COVID-19 are enormous and we have tried to control for confounding factors happened in 2022, we might not be able to address these issues completely. Thus, the results should be interpreted as correlations, rather than* *causal effects.*

5. Ensure Consistency in Reported Results: Correct the discrepancies in percentage/point changes between the main text summary (page 20) and the regression tables (Table 4).

**Response:**

Thank you very much for this comment which we totally agree. We have revised the conclusion with the new results and ensure the consistency in reporting the results.

- Revised text: Paragraph 1 of section “Conclusions and policy implications” (Pages 20 and 21)

*The introduction of physical distancing measures (i.e., lock down) and the disruptions in production and supply chains caused by the COVID-19 pandemic have resulted in reduced livelihoods, increased food insecurity, increased poverty, and increased inequality. Given the limited evidence on the impacts of the COVID-19 at the micro level, we use panel data for Thailand and Vietnam to examine (i) the correlation and heterogeneous effects of the COVID-19 pandemic on household income, income inequality, and poverty and (ii) the distributional effects of the pandemic on household income. The results of fixed-effects estimations show that the COVID-19 has a negative correlation with household income, while it has a positive correlation with the Gini coefficient of income inequality, absolute poverty, and multidimensional poverty. In particular, compared to the before-the-COVID-19 period, the COVID-19 has resulted in a decrease of the daily per capita income of rural households by 19.67%. The COVID-19 increases the Gini coefficient of income inequality by 0.013 and the percentages of household living under absolute poverty and multidimensional poverty by 7.1% compared to the before-the-COVID-19 period.*

6. Correct Interpretation of Education Interaction: Ensure the text on page 17 accurately reflects that education reduces the poverty impact of COVID-19, consistent with Table 5.

**Response:**

We are thankful that you helped us pointing out this silly mistake. The correct reading of the sentence should be “*In contrast, in the context of the COVID-19, average schooling years of adult members have positive effects on household’s daily per capita income and the Gini coefficient of income inequality, but it has a negative impact on absolute income poverty and multidimensional poverty”.*

**3. Comments and suggestion from the Reviewer #2**

Reviewer #2: This paper examines the correlation between income, inequality, and poverty at the provincial level for Thailand and Vietnam, using panel data and focusing on the year 2022, which the authors treat as the COVID-19 year. The analysis relies on a fixed-effects model, followed by an exploration of heterogeneous effects. The main conclusion is that the year 2022 is positively correlated with the Gini coefficient and poverty, and negatively correlated with income.
While I appreciate the motivation of the paper and its objectives, I have concerns about the identification strategy.

**Response:**

Thank you very much for your valuable comments and suggestions which helped us improve our manuscript. We have acknowledged your help in the acknowledgements section. As you can see in this revised version, all your comments and suggestions have been carefully considered and addressed. Especially, we agree with your comment on including more variables at the provincial level. Thank you very much for the comments!

1. In particular, I am not convinced that treating 2022 as a proxy for COVID-19 captures the pure effect of the pandemic on socioeconomic variables. Other policies and shocks that occurred in 2022 may confound this proxy. To make the analysis cleaner, I suggest adding provincial-level controls such as unemployment and the percentage of households receiving social aid.

**Response:**

Thank you very much for your comment which we follow. First, in this revised version, we have included two variables at the provincial levels, namely the unemployment rate and share of rural population. We could not obtain the information about the number or the share of households receiving social aids at the provincial level in two countries. However, we believe that the two variables that we use, to a certain extent, could reflect the socio-economic conditions in these provinces. Second, in our study, except for the heterogenous and distributional effects, we use the term “correlation” instead of “effects” of the COVID-19 period. Last, we acknowledge this issue and have included a sentence in the limitation regarding this issue.

2. I also recommend including a dedicated limitations section. In this section, the authors could explicitly discuss the concerns raised above and acknowledge other caveats that may affect their results.

**Response:**

Thank you very much for your comment which we follow. We have included a paragraph in the “Conclusions and policy implications” to acknowledge the limitation of our current study as you can also see below.

***- Added text: Paragraph 5 of the “Conclusions and policy implications” (Pages 22 – 23)***

*Although our study has provided some useful insights, it is not free from limitations. First, we use the panel data which have inconsistent time gaps between panel waves. There was a large time gap between panel waves which might not well reflect the short-term change of household’s livelihood before, during, and after the COVID-19. Hence, the use of panels with shorter time gaps is strongly recommended in future studies. Second, although the attrition rate of our sample is low, due to the fact that one province in Vietnam was excluded in the 2022 survey, the results should be interpreted strictly for the reduced sample. Third, the Gini coefficients of income inequality in our model are calculated at the provincial levels, hence the results regarding the correlation of the COVID-19 with the Gini coefficient should be interpreted with caution. Last, even though the impacts of the COVID-19 are enormous and we have tried to control for confounding factors happened in 2022, we might not be able to address these issues completely. Thus, the results should be interpreted as correlations, rather than causal effects.*

3. In addition, the robustness of the findings would be strengthened by applying the reweighting method of DiNardo, Fortin, and Lemieux (1996). In particular, the authors could reweight the provincial income distribution using observables from the 2016 wave as a counterfactual benchmark.

**Response:**

Thank you for your comment. The monetary values in our data were adjusted to inflation and 2005 prices. The values could be used for benchmarking. Our model which estimates the distributional effects using the Unconditional Quantile Regression (UQR) is actually similar to the approach of DiNardo et al. (1996) as explained by Firpo et al. (2007, 2009). More importantly, the application of the reweighted provincial income using observables from the 2016 wave might make the fixed-effects estimation model become more complicated and redundant in the UQR estimation.

***Cited documents:***

Firpo, S., Fortin, N. M., & Lemieux, T. (2007). Decomposing Wage Distributions using Recentered Influence Function Regressions. Available from: <https://www.economics.uci.edu/files/docs/micro/f07/lemieux.pdf> (Accessed: 17 Oct 2025)

Firpo, S., Fortin, N. M., & Lemieux, T. (2009). Unconditional quantile regressions. *Econometrica*, *77*(3), 953-973.

4.1 Finally, the paper would benefit from a careful proofreading. For example, in the introduction the authors write “… further deepened the inequality …”. Inequality of what? Clarifying such statements would improve readability.

**Response:**

We thank you for your comment. The “inequality” which we mention in this sentence is referred to the “inequality” of income, consumption, and economic growth in the first paragraph and from cited documents as “[2, 3, 4, 21, 22]”. Nevertheless, we agree with you and have conducted a careful proofreading of the manuscript to clarify vague statements like this.

4.2 In terms of technical presentation, I suggest:

In equation (1), use the subindex w for provinces.

In equation (2), present definitions for indices i and w immediately after the equation.

**Response:**

Thank you for your comment. We have included a subindex “*p*” for indicating province in all equations (we keep the subindex *w* for the wave). The revision of Equation 1 can be found below (as Revised Text 1). Regarding Equations (2) and (3), we have included provincial variables as your comment #1. We have now three subindexes including *i* for household, *p* for province, and *w* for survey wave. As recommended, we have revised the explanation part of the Equation 2 to move the definitions for subindexes right after introducing the Equation. Our revision can be found below (as Revised Text 2).

***- Revised text 1: Equation 1 of the subsection “Measurement of income inequality” (Page 8)***

*In particular, the Gini coefficient of province p (*$G_{p}$*) is calculated as follows:*

$G_{p}=1-\sum_{z=1}^{n} (X_{z}-X_{z-1})(Y_{z}+Y_{z-1})$	*(1)*

***- Revised text 2: Explanation on Equation 2 of the subsection “Investigating the correlation and the heterogenous effects of the COVID-19 on household income, inequality, and poverty” (Pages 13 and 14)***

*The household fixed-effects model can be specified as follows:*

$\boldsymbol{Y}_{\boldsymbol{ipw}}= \beta_{0}+\beta_{1}\boldsymbol{COVID}_{\boldsymbol{w}}+\beta_{2}\boldsymbol{X}_{\boldsymbol{ipw}}+\beta_{3}\boldsymbol{P}_{\boldsymbol{pw}}\boldsymbol{+}h_{ip}+\omega_{ipw}$	*(2)*

*where* $\boldsymbol{Y}_{\boldsymbol{ipw}}$ *refers to indicators of household i from province p at wave w including (i) daily per capita income, (ii) Gini coefficient of income inequality, (iii) absolute income poverty, and (iv) multidimensional poverty.* $\boldsymbol{COVID}_{\boldsymbol{w}}$ *is a binary variable (COVID-19 period (i.e., wave of 2022) = 1; otherwise = 0).* $\boldsymbol{X}_{\boldsymbol{ipw}}$ *is a group of household variables. In this study, we control for important indicators of household livelihood which have been widely used in livelihood and poverty-related studies [12, 45], namely age of head, gender of head, household size, number of adults in household, number of elderly members in household (as household’s demographic characteristics), ethnicity of head, membership in political and social organization (as household’s social capital), share of farm laborers, schooling years of head, average schooling years of adult members (as household’s human capital), land area, and asset-poor household (as household’s wealthy level). We further control for a member contracted to the COVID-19 and exposure to any shocks in the past 12 months since these events might influence household income.* $\boldsymbol{P}_{\boldsymbol{pw}}$ *are province level’s variables, namely unemployment rate and the share of rural population.* $h_{ip}$ *is the household fixed-effects and* $\omega_{ipw}$ *is the unobserved time-varying idiosyncratic errors.*
