# Supplementary material for: Households’ poverty and inequality after the COVID-19: Insights from panel data of face-to-face surveys in Southeast Asia
Source: PLoS One. 2026 Jan 30;21(1):e0341648. doi: 10.1371/journal.pone.0341648 (PMC12922772; doi:10.1371/journal.pone.0341648)
Supplement: Attachment — Submitted filename: 2. Response_to_editor.docx [file pone.0341648.s014.docx]

**Response to the Editor’s comments and suggestions**

*Please note: the comments and suggestions of the Editor are in blue, our responses in black, the revised/newly added text in red, and the STATA commands in green.*

**1. Comments and suggestion from the Editor**

Thank you very much for your revisions, which have significantly improved the paper.

However, I would appreciate it if the authors could provide a more in-depth response to the third comment from Referee 2, as their current answer lacks sufficient accuracy and clarity. Specifically, while both the DiNardo, Fortin, and Lemieux (1996) reweighting approach and Unconditional Quantile Regression (Firpo et al., 2009) are motivated by distributional analysis, they are distinct methods that address different objectives, and they are not equivalent.

The DFL approach constructs counterfactual income distributions by reweighting observables, while UQR estimates marginal effects on unconditional quantiles. Therefore, the claim that UQR is "similar" to DFL and renders reweighting redundant is not fully justified. Furthermore, it is entirely feasible to apply the DFL reweighting and then estimate UQR models on the reweighted sample.

I would therefore request that the authors either implement the suggested reweighting exercise as a robustness check or offer a clearer, technically sound explanation of why this approach might not be suitable for their specific empirical context. They should also explicitly address the conceptual differences between the two methods to avoid any confusion.

**Response:**

Thank you very much for your consideration and further comments. Your comment on the difference between the DFL and UQR approaches is totally correct. Please allow us to clarify our response to comment #3 of the Reviewer 2 in the previous round that “Our model which estimates the distributional effects using the Unconditional Quantile Regression (UQR) is actually similar to the approach of DiNardo et al. (1996) as explained by Firpo et al. (2007, 2009)”. To estimate the UQR, we use the “rifhdreg” command from Rios-Avila (2020). This model could calculate the recentered influence function (RIF) in estimating the UQR (Firpo et al., 2009) and other distributional statistics as mentioned in (Firpo et al., 2018). According to Rios-Avila (2020), the command fits the RIF-regression model in two steps. “First, it estimates the RIF for the distributional statistic of interest. Second, it uses the estimated RIF as dependent variable and fits the RIF model”. In Firpo et al. (2018), it is explained that “the first stage consists of decomposing the distributional statistic of interest… The idea of the first stage is thus very similar to DiNardo et al. (1996)” (pp. 2). Therefore, we relied on these details to mention the similarity of the two approaches, not to indicate they are the same or equivalent. We are grateful for your comment on this issue to avoid the confusion.

We follow your suggestion of estimating the reweighting (i.e, the weights) and apply them into the UQR models. We employ the “reldist” command and “pdf” as relative density from Jann (2020) (see also Jann (2021) for further explanation on the command and approach).

*- Step 1: Creating balancing weights*

There are two ways to generate the balancing weights. The first can be generated through:

global control treated age gender ethnic hh_size no_adult no_elderly socio_member share_farmlab edu_year mean_edu_hh shock_exposure area_perca asset_poor prov_unemploy prov_ruralpop

reldist pdf dai_inc_per, by(time) balance($control, generate(weight_time1))

The weights could be generated, but the balancing is not so good:

tabstat $control [aw=weight_time1], by(time) nototal

Alternatively, the entropy balancing (as “eb” added into the syntax) can be applied as:

reldist pdf dai_inc_per, by(time) balance(eb: $control, generate(weight_time2))

tabstat $control [aw=weight_time2], by(time) nototal

We can see that the balancing is much better now. Hence, we will apply the weights produced from the “entropy balancing” method to our model.

*- Step 2: Running the estimation of the UQR model using the weights from the step #1*

Next, we include the balancing weights from the previous step in the estimation of the UQR models. The results of these estimations are presented in Table 1 below (as S8 Table in the manuscript). Overall, the negative effects of the COVID-19 on household income can still be observed. One difference is that the negative effects on the income of households in the 90^th^ quantile group are now significant. However, the effects on income of households in the 10^th^ and 25^th^ quantile groups remain the largest with the reduction of 111% and 56%, respectively. Hence, as a robustness check, we have added these details to the sub-section “The distributional effects of the COVID-19 on household income” you can also find as below.

***- Added text: Paragraph 2 of the “The distributional effects of the COVID-19 on household income” (Pages 20 - 21)***

*As a robustness check, we employ the relative distribution approach from [62] to estimate the balancing weights of the covariates before and after the COVID-19 (see [63] for further explanation on the approach). We use the “reldist” command from STATA with “pdf” as the relative density and the entropy balancing method to generate the balancing weights. These weights are, then, applied to the estimations of UQR models. The results of these estimations on the distributional effects on household income using balancing weights are presented in S8 Table in the supplementary information. Overall, the negative effects of the COVID-19 on household income can still be observed. One difference is that the negative effects on the income of households in the 90th quantile group are now significant. However, the largest effects are still on the income of households in the 10th and 25th quantile groups with the reduction of 111% and 56%, respectively. The negative effects of the COVID-19 on household income’s distribution remain consistent after applying the balancing weights.*

***Cited documents:***

Firpo, S. P., N. M. Fortin, and T. Lemieux. (2009). Unconditional quantile regressions. *Econometrica*, 77, 953-973. <https://doi.org/10.3982/ECTA6822>

Firpo, S. P., N. M. Fortin, and T. Lemieux. (2018). Decomposing wage distributions using recentered influence function regressions. *Econometrics*, 6, 28. <https://doi.org/10.3390/econometrics6020028>

Jann, B. (2020). reldist: Stata module for relative distribution analysis. Available from <https://ideas.repec.org/c/boc/bocode/s458775.html>

Jann, B. (2021). Relative distribution analysis in Stata. *The Stata Journal*, *21*(4), 885-951. <https://doi.org/10.1177/1536867X211063147>

Rios-Avila, F. (2020). Recentered influence functions (RIFs) in Stata: RIF regression and RIF decomposition. *The Stata Journal*, *20*(1), 51-94. <https://doi.org/10.1177/1536867X20909690>

**Table 1. Robustness check of the** **distributional effects of the COVID-19 on household income using balancing weights**

|  | Daily per capita income (PPP$) | | | | |
| --- | --- | --- | --- | --- | --- |
|  | 10^th^ | 25^th^ | 50^th^ | 75^th^ | 90^th^ |
| COVID-19 period^†^ | -0.771^**^ | -1.003^***^ | -0.976^*^ | 0.385 | -5.131^***^ |
|  | (0.355) | (0.377) | (0.519) | (1.120) | (1.258) |
| Member contracted to the COVID-19^†^ | -0.115 | 0.128 | -2.795^*^ | -3.038 | 2.626 |
|  | (0.567) | (0.592) | (1.507) | (2.184) | (2.550) |
| Age of head | 0.016 | 0.004 | -0.025 | -0.030 | 0.116^***^ |
|  | (0.013) | (0.019) | (0.016) | (0.037) | (0.043) |
| Male head^†^ | 0.280 | 0.309 | 0.429 | 0.374 | 0.648 |
|  | (0.177) | (0.231) | (0.282) | (0.394) | (0.892) |
| Ethnic majority^†^ | -0.110 | 0.373 | 0.944^***^ | 0.408 | 0.017 |
|  | (0.362) | (0.500) | (0.279) | (0.341) | (0.906) |
| Household size | -0.218^*^ | -0.323^***^ | -0.708^***^ | -0.924^***^ | -2.419^***^ |
|  | (0.119) | (0.083) | (0.098) | (0.181) | (0.314) |
| Number of adults | 0.228 | 0.486^***^ | 1.078^***^ | 1.670^***^ | 2.183^***^ |
|  | (0.167) | (0.127) | (0.141) | (0.273) | (0.526) |
| Number of elderly members | 0.202 | 0.461^*^ | 0.997^***^ | 1.536^**^ | -0.297 |
|  | (0.266) | (0.270) | (0.321) | (0.711) | (0.770) |
| PSO member^†^ | 0.537^***^ | 0.676^***^ | 0.851^*^ | 1.479^*^ | -2.964^**^ |
|  | (0.191) | (0.238) | (0.459) | (0.869) | (1.194) |
| Share of farm laborers | -0.009^***^ | -0.014^***^ | -0.025^***^ | -0.025^***^ | -0.058^***^ |
|  | (0.003) | (0.003) | (0.004) | (0.007) | (0.012) |
| Schooling years of head | 0.025 | 0.056^**^ | 0.134^***^ | 0.279^***^ | 0.767^***^ |
|  | (0.033) | (0.028) | (0.048) | (0.093) | (0.187) |
| Mean schooling years of adult members | 0.046 | 0.072^**^ | 0.218^***^ | 0.182^**^ | 0.538^***^ |
|  | (0.030) | (0.030) | (0.045) | (0.071) | (0.151) |
| Shock exposure^†^ | -0.131 | -0.059 | 0.132 | -0.356 | -0.788 |
|  | (0.159) | (0.195) | (0.268) | (0.481) | (0.691) |
| Land area per capita | 0.063 | 0.376^***^ | 0.818^***^ | 1.520^***^ | 4.011^***^ |
|  | (0.077) | (0.093) | (0.120) | (0.229) | (0.538) |
| Asset poor^†^ | -0.674^***^ | -1.081^***^ | -2.330^***^ | -2.146^***^ | -3.029^***^ |
|  | (0.239) | (0.258) | (0.219) | (0.294) | (0.658) |
| Province’s unemployment rate | -0.144^*^ | -0.218^**^ | -0.497^***^ | -0.614^**^ | 0.201 |
|  | (0.081) | (0.086) | (0.143) | (0.291) | (0.406) |
| Province’s share of rural population | -0.041^**^ | -0.037^***^ | -0.041^*^ | -0.036 | -0.095 |
|  | (0.016) | (0.013) | (0.021) | (0.031) | (0.085) |
| Constant | 3.812^**^ | 4.558^***^ | 7.722^***^ | 10.060^***^ | 15.135^**^ |
|  | (1.649) | (1.341) | (2.053) | (3.127) | (7.655) |
| Number of observations | 10068 | 10068 | 10068 | 10068 | 10068 |
| F (17,361) | 4.005 | 15.099 | 83.176 | 56.853 | 20.437 |
| Prob. > F | 0.000 | 0.000 | 0.000 | 0.000 | 0.000 |
| R^2^ | 0.075 | 0.130 | 0.200 | 0.177 | 0.096 |
| Sample mean RIF | 0.693 | 1.773 | 3.931 | 7.803 | 12.432 |
| Impact magnitude on household’s daily per capita income | -111.33% | -56.58% | -24.83% | Not significant | -41.27% |

Note: Robust standard errors clustered at village level in parentheses; ^†^: Dummy; ^∗∗∗^ *p*<0.01, ^∗∗^ *p*<0.05, ^∗^ *p*<0.1.
